# Prevalence of low Back pain among adolescents in relation to the weight of school bags

**DOI:** 10.1186/s12891-019-2398-2

**Published:** 2019-01-22

**Authors:** Fatemah Akbar, Muneera AlBesharah, Jumana Al-Baghli, Farah Bulbul, Dana Mohammad, Bann Qadoura, Abdullah Al-Taiar

**Affiliations:** Department of Community Medicine and Behavioral Sciences, Faculty of Medicine, Kuwait University, Box: 24923, 13110 Safat, Kuwait

**Keywords:** Low Back pain, Adolescents, School bags, Kuwait

## Abstract

**Background:**

The association between the weight of school bag and Low Back Pain (LBP) amongst students remains under intense debate worldwide. This study aimed to estimate the prevalence of LBP amongst public high school students (14 to 19 years) in Kuwait and to investigate the association between LBP and the weight of school bags.

**Methods:**

An analytical cross-sectional study using multistage cluster random sampling with probability proportional to size was conducted on a total of 950 public high school students from all governorates. Data on LBP were collected through face-to-face interviews using a structured questionnaire. A 0–10 Numeric Pain Rating Scale was used to rate the intensity of LBP. The students’ height and weight in addition to the weight of their school bags were measured using appropriate weight and height scales. Logistic regression was used to investigate the association between the weight of school bags and LBP while adjusting for potential confounders.

**Results:**

The estimated lifetime, 6-month, and 1-month prevalence of LBP were 70.3% (95% CI: 67.30–73.21%), 49.1% (95% CI: 45.83–52.28%), and 30.8% (95% CI: 27.81–33.78%) respectively, with significantly higher prevalence amongst females compared to males (*p* < 0.001). The absolute weight of school bag was not significantly associated with LBP neither in univariable nor multivariable analysis. The relative weight of school bag (as a percentage of the body weight) was significantly associated with LBP in univariable analysis but not in multivariable analysis. The perceived heaviness of school bag, however, was found to be significantly associated with LBP throughout the analysis (*p* < 0.001).

**Conclusion:**

In conclusion, LBP amongst high school students in Kuwait seems to be very common with a prevalence resembling that of high-income countries. Our data suggest that the perceived heaviness of school bag is far more important than the actual bag weight. Current recommendations about the weight of school bags, which are not supported by evidence, should be revised to take into account the students’ perceived heaviness of school bag.

## Background

Although Low Back Pain (LBP) is not a life-threatening condition, it is a major cause of absenteeism from work and loss of productivity, in addition to severe implications on the quality of life [1]. In The Global Burden of Disease study, LBP ranked highest in terms of years lost due to disability, and sixth in terms of disability-adjusted life years [2]. LBP among adolescents associates with school absenteeism and loss of educability [3] in addition to the impact on quality of life. There is a considerable geographical variation in the prevalence of LBP among adolescents. As an example, the lifetime prevalence in European countries such as the United Kingdom [4] and Norway [5] was reported to be 55 and 63% respectively, which is higher than lifetime prevalence in African countries such as Tunisia (28.4%) [6] and Mozambique (28%) [7]. A previous study in Kuwait, reported the lifetime prevalence of LBP amongst high school adolescents to be 57.8% [8]. Although the results of the studies remain controversial, many studies showed that female adolescents experience more LBP than males [9–11].

Heavy school bags have been suspected to be a predisposing factor for LBP amongst school adolescents. Recently, it has been recommended by several associations, such as The Ontario Chiropractic Association, The American Occupational Therapy Association, and The American Academy of Pediatrics, that the school bag weight should not exceed 10 to 15% of the child’s body weight; otherwise, the student will be at a greater risk of LBP [12]. This recommendation; however, was not evidence-based and that most studies found no link between the measured weight of school bag and LBP [13–15]. This study aimed to estimate the prevalence of LBP amongst public high school students (14 to 19 years) in Kuwait and to investigate the association between LBP and the weight of school bag.

## Methods

This study was approved by The Ethics Committee at Health Science Center, Kuwait University (Ref: 106–12/03/2017). We also obtained permissions from The Ministry of Education and high school principals. Written informed consent was taken from each student before initiating the interview.

### Study design, study population and sampling technique

An analytical cross-sectional study was conducted on public high schools students, who are typically in the age between 14 and 19 years. The public education system in Kuwait follows a single-sex education hence boys and girls are in separate schools. We obtained a list of all public high schools along with the number of students in each school from The Ministry of Education. We stratified schools by gender and used a multistage cluster random sampling to select a representative sample of students at public high schools. In each governorate, one school for boys and one school for girls was selected using probability proportional to size sampling technique (12 schools were selected in total). In this method, schools with large number of students were given higher probability to be selected compared to schools with small number of students. We distribute the sample between different governorates based on the relative size of each governorate, which was judged by the total number of students in each governorate compared to the total number of students in the whole country (proportional allocation).

### Data collection

Data on LBP were collected by face-to-face interview using a structured questionnaire. The questionnaire was developed in English and then translated into Arabic. The Arabic version of the questionnaire was then back-translated to English by two independent individuals who were not involved in the study. The original English and the back-translated were then compared. The final Arabic version of the questionnaire was later pre-tested on 20 students to check for ambiguity and estimate the time of the interview.

The questions on LBP were developed after an extensive literature review of studies that investigated LBP among adolescents. A photo card was used to show each student the exact location of LBP. Questions were developed to gather data on the period prevalence of LBP, including the lifetime, 6-month, and 1-month prevalence. Lifetime prevalence was evaluated by the question “Have you ever felt low back pain that lasted a day or longer? (Yes, No, I don’t remember)”, while 6-month period prevalence was gauged by the question “Have you felt low back pain that lasted a day or longer in the last six months? (Yes, No, I don’t remember)”. Questions about the frequency of LBP, its impact on daily life activities, treatment needed, absenteeism from school due to LBP, previous back injuries and relatedness of LBP to menstrual cycle (amongst females) were included. A 0–10 Numeric Pain Rating Scale, which has not been validated in our setting, was used to rate the intensity of LBP.

The weight (kg) of every student’s bag was measured using a portable digital luggage scale (SAFEWAY^R^) that was calibrated before each use. A hook was attached to the highest point of the bag then lifted off the ground until a steady reading was noted. The perceived heaviness of school bags was evaluated by asking the students the following question: “How would you describe the weight of your school bag? (Light, Normal, Heavy, Too heavy)”. The data collection tool also included questions on the number of school bags each student carries, type of the school bag (using a photo card) and the way each student carries his/her bag (using a photo card). Students were also asked about walking to schools and the use of lockers to reduce the weight of their bags. The height of each student was measured using a portable stable stadiometer (SECATM^R^) to the nearest 0.1 cm. Each student was asked to remove his/her shoes and stand with heels together, back and head straight, against a wall, making sure the horizontal bar of the stadiometer was at the level of the student’s head. Weight was measured using a digital weight scale (Beurer^R^ GS 19) to the nearest 0.1 kg after removing shoes and any heavy clothing.

### Statistical analysis

Data were analyzed using Statistical Package for Social Sciences (SPSS) version 24. Body Mass Index was calculated as weight (kg)/height (m)^2^. Obesity and overweight were defined according to WHO growth charts. 95% CIs for the prevalence were calculated using exact binomial distribution. We used unconditional logistic regression to investigate the association between the weight of school bags and LBP. The absolute bag weight was fitted as a continuous variable and then was categorized into tertiles and fitted as an indicator variable. Similarly, the bag weight was calculated as a percentage of the body weight and then fitted firstly as a continuous variable and secondly as an indicator variable after it was grouped into tertiles. Variables that showed an association with LBP at 20% level of significance in univariable analysis were considered in multivariable analysis. Confounders were grouped into socio-demographic factors, bag-related factors and lifestyle factors. Each group of confounders were introduced sequentially to the model and the impact of this on the association between the weight of school bag and LBP was noted. The statistical significance of the association between the weight of school bag and LBP was assessed using likelihood ratio test, which compares models with and without the variable.

## Results

### Description of the study group

Of 958 students who were invited to participate, no student refused but one student terminated the interview promptly and was excluded. Seven students were excluded from the analysis because calculating their age from the date of birth showed they were actually over 20 years old. The analysis below comprises 950 students. Table 1 shows the socio-demographic factors of the study participants. The mean (SD) age of the participating was 16.7 (1.0) years and 536 (56.4%) were males.

**Table 1:** Socio-demographic characteristics of 950 public high school students in Kuwait, 2017

Characteristic	*n*	(%)
Gender		
Males	536	(56.4)
Females	414	(43.6)
Age in years, mean (SD)	16.7	(1.0)
Nationality		
Kuwaiti	764	(80.4)
Non-Kuwaiti	186	(19.6)
Governorate		
Al-Jahra	173	(18.2)
Hawalli	156	(16.4)
Al-Asimah (Capital)	173	(18.2)
Al-Farwaniyah	157	(16.5)
Al-Ahmadi	184	(19.4)
Mubarak Al-Kabeer	107	(11.3)
School grade of students		
10	375	(39.5)
11	242	(25.5)
12	333	(35.1)
Father’s highest level of education		
No formal education	3	(0.3)
Elementary/middle school degree	110	(11.6)
High school diploma	244	(25.7)
Diploma/university degree	420	(44.2)
Unknown	173	(18.2)
Mother’s highest level of education		
No formal education	34	(3.6)
Elementary/middle school degree	111	(11.7)
High school diploma	235	(24.7)
Diploma/university degree	426	(44.8)
Unknown	144	(15.2)

### Prevalence of LBP

Table 2 shows the lifetime, 6-month, and 1-month prevalence of LBP. These were estimated to be 70.3% (95% CI: 67.3–73.2%), 49.1% (95% CI: 45.8–52.3%), and 30.8% (95% CI: 27.8–33.8%) respectively. The lifetime, 6-month, and 1-month prevalence were not significantly different between Kuwaiti and non-Kuwaiti participants (*p* = 0.183, *p* = 0.736 and *p* = 0.512, respectively). The lifetime, 6-month, and 1-month prevalence were consistently higher among females compared to males (*p* < 0.001, p < 0.001, p < 0.001, respectively).Of the 293 students with LBP last month, 21.5% were absent in one or more school days during last month.

**Table 2:** Prevalence of low back pain amongst 950 public high school students in Kuwait, 2017

Question	*n*	(%)
Have you ever felt low back pain that lasted a day or longer?		
Yes	668	(70.3)
Have you felt low back pain that lasted a day or longer in the last 6 months?		
Yes	466	(49.1)
Have you felt low back pain that lasted a day or longer in the past month?		
Yes	293	(30.8)
How many times did you feel this back pain in the last month? (*n*=293)		
Once	39	(13.3)
2-3 times	120	(41.0)
4-5 times	57	(19.5)
6 times or more	77	(26.3)
Severity of LBP^a^, mean (SD)^b^	5.48	(1.6)

^a^ Low Back Pain. ^b^ Data were collected using numeric pain rating scale from 0 to 10

### Association between LBP and the weight of school bags

In order to investigate the association between LBP and the weight of school bags, we used unconditional logistic regression. In this analysis, 6-month prevalence of LBP was used as the binary outcome. Those who responded “Do not remember” for the question about back pain in the last 6 months were excluded from this analysis and thus the analysis below comprised 918 students.

The crude association between LBP and the weight of school bag (the exposure) as well as other covariates is demonstrated in Table 3. Table 4 shows the association between the relative weight of school bag (as a percentage of total body weight) and LBP before and after adjusting for various confounders. The relative weight of school bag was significantly associated with LBP in univariable analysis but not after adjusting for potential confounders. In fact adjusting for the perceived heaviness of school bag was sufficient to show that there is no association between the relative weight of school bags and LBP. Because this could be due to collinearity, we repeated the analysis above excluding the perceived heaviness of school bag and also found no association between LBP and the relative weight of school bags after adjusting for potential confounders. The same analysis was conducted using the absolute bag weight (not as a percentage of body weight); and we found no association between bag weight and LBP before or after adjusting for potential confounders. We also analyzed the data using the absolute bag weight as a continuous variable; and found no association between the absolute bag weight and LBP in multivariable analysis. Finally, we repeated the analysis after categorizing the relative weight of school bag into three groups (≤10% of body weight, > 10% to ≤15% of body weight, and > 15% of body weight [12]) and found no association between the relative weight of school bag and LBP in multivariable analysis. These findings remained unchanged when we exclude 40 participants who reported previous back injury. Throughout the analysis, the only factors that remained consistently associated with LBP were gender and how the students perceived the heaviness of their bag.

**Table 3:** Association between low back pain and the weight of school bag and other risk factors among 918 public high school students in Kuwait, 2017

Factor	Odds ratio of LBP (6-month)	
	OR [95% CI]	*p*-value^1^
Bag weight in kg		
≤ 5.32	1.00 [Reference]	0.620
5.33–7.35	1.17 [0.85–1.61]	
≥ 7.36	1.10 [0.80–1.50]	
Bag weight as percent of body weight		
< 10%	1.00[Reference]	0.041
10 to 15%	1.42 [1.07–1.90]	
≥ 15%	0.95 [0.62–1.10]	
Gender		
Males	1.00 [Reference]	<0.001
Females	2.17 [1.66–2.82]	
Age (years)	0.98 [0 .87–1.11]	0.792
Nationality		
Kuwaiti	1.00 [Reference]	0.790
Non-Kuwaiti	1.04 [0.76–1.44]	
Governorate		
Al-Jahra	1.00 [Reference]	0.102
Hawalli	0.96 [0.62–1.49]	
Al-Asimah (Capital)	1.11 [0.72–1.71]	
Al-Farwaniyah	0.76 [0.49–1.18]	
Al-Ahmadi	0.62 [0.41–0.95]	
Mubarak Al-Kabeer	0.90 [0.55–1.46]	
Father’s education		
No formal education/ middle school	1.00 [Reference]	0.093
High school	0.89 [0.56–1.40]	
Diploma/university degree	1.15 [0.75–1.75]	
Unknown	0.74 [0.46–1.20]	
Mother’s education		
No formal education/middle school	1.00 [Reference]	0.332
High school	1.14 [0.74–1.73]	
Diploma/university degree	1.32 [0.90–1.95]	
Unknown	1.00 [0.63–1.60]	
Perceived heaviness of school bag^1^		
Light	1.00 [Reference]	< 0.001
Normal	1.24 [0.70–2.18]	
Heavy	1.98 [1.13–3.50]	
Very heavy	4.20 [2.19–8.06]	
Carrying a school bag ≤4 days or never^3^	1.00 [Reference]	< 0.001
Carrying a school bag every day	2.84 [1.72–4.68]	
Number of bags taken to school		
None	1.00 [Reference]	< 0.001
One	2.95 [1.76–4.97]	
Two	4.14 [1.56–11.00]	
More than two	8.00 [.79–81.25]	
The way of carrying school bag^4^		
On two shoulders	1.00 [Reference]	0.384
On one shoulder	1.02 [0.72–1.43]	
Others	0.54 [0.98–1.33]	
Using lockers to reduce the weight of school bag		
Yes	1.00 [Reference]	0.156
No	0.46 [0.16–1.34]	
Walking to/from school per week^5^		
None	1.00 [Reference]	0.41
1–8 times	0.78 [0.50–1.21]	
Every day (10 times)	1.21 [0.68–2.16]	
Playing sports per week		
None	1.00 [Reference]	0.97
Once	1.03 [0.69–1.55]	
2–3 times	1.09 [0.76–1.58]	
More than 3 times	1.06 [0.73–1.55]	
Watching TV/ using computer per day		
≤ 2 h	1.00 [Reference]	0.012
3–5 h	1.33 [0.97–1.82]	
≥ 6 h	1.76 [1.16–2.68]	
Using iPad/ playing videogames per day		
≤ 2 h	1.00 [Reference]	0.492
3–5 h	1.21 [0.88–1.66]	
≥ 6 h	0.99 [0.66–1.49]	
Studying/ doing homework/ reading per day		
≤ 2 h	1.00 [Reference]	0.483
3–5 h	1.09 [0.79–1.48]	
≥ 6 h	1.43 [0.78–2.62]	
Favorite place for studying at home		
Bed	1.00 [Reference]	0.963
Desk	0.94 [0.69–1.28]	
Floor	1.04 [0.72–1.50]	
Couch	0.95 [0.61–1.49]	
Other	0.63 [0.10–3.84]	
Cigarette smoking		
Non-smoker	1.00 [Reference]	0.060
Ex-smoker	0.64 [0.38–1.09]	
Current smoker	0.71 [0.52–1.01]	
Shisha smoking		
Non-smoker	1.00 [Reference]	0.410
Ex-smoker	1.45 [0.64–3.26]	
Current smoker	0.76 [0.43–1.35]	
GPA of the participant		
Less than 74%	1.00 [Reference]	0.277
74%- less than 85%	1.16 [0.84–1.95]	
≥ 85%	1.29 [0.94–1.77]	
BMI categories^6^		
Normal/underweight	1.00 [Reference]	0.587
Overweight	1.20 [0.85–1.70	
Obese	0.94 [0.70–1.27]	
Underweight	0.88 [0.33–2.31]	

^1^*p*-values were generated using likelihood ratio test

^2^as reported by the students and 78 students did not have school bags

^3^78 students who did not have school bags are included in the reference group

^4^data were collected using a photo card and those who did not have school bags were excluded in this analysis

^5^going and coming from school is two times

^6^weight and height were measured and WHO growth charts were used to calculate z-score

**Table 4:** Association between bag weight (as a percentage of body weight) and low back pain before and after adjusting for potential confounders

Bag weight (% of body weight)	Model 1 OR [95%CI]	Model 2 OR [95%CI]	Model 3 OR [95%CI]	Model 4 OR [95%CI]
< 7.3%	1.00 [Reference]	1.00 [Reference]	1.00 [Reference]	1.00 [Reference]
7.3 to < 11%	1.32 [0.96–1.82]	1.09 [0.78–1.52]	0.80 [0.56–1.15]	0.79 [0.55–1.14]
≥ 11%	1.49 [1.08–2.04]	1.25 [0.90–1.76]	0.85 [0. 59–1.24]	0.84 [0.58–1.23]
*p*-value	0.041	0.390	0.488	0.458

OR: odds ratio; **Model 1**: unadjusted; **Model 2**: adjusted for socio-demographic factors (gender, governorate, education of the father); **Model 3**: adjusted for variables in the Model 2 in addition to the perceived heaviness of school bags, number of days the students carry their bag to schools and number of bags taken to schools; **Model 4**: adjusted for variables in Model 3 in addition to smoking and hours per day spent on watching TV/using computer

Table 5 shows the association between the perceived heaviness of school bag and LBP before and after adjusting for potential confounders. There was highly significant association between how the students perceived heaviness of their school bag and LBP in all models. Those who described their school bag as “heavy” or “very heavy” had higher odds of LBP. We repeated this analysis while categorizing those with no school bags (the question about the weight of their school bag was not applicable) in the reference group; and the findings remain practically unchanged.

**Table 5:** Association between the perceived heaviness of school bags and low back pain before and after adjusting for potential confounders

Perceived heaviness of school bag*	Model 1 OR [95%CI]	Model 2 OR [95%CI]	Model 3 OR [95%CI]	Model 4 OR [95%CI]
Light	1.00 [Reference]	1.00 [Reference]	1.00 [Reference]	1.00 [Reference]
Normal	1.23 [0.70–2.18]	1.19 [0.67–2.12]	1.19 [0.66–2.12]	1.20 [0.67–2.15]
Heavy	1.98 [1.13–3.50]	1.71 [0.95–3.04]	1.68 [0.94–3.00]	1.65 [0.92–2.96]
Very heavy	4.20 [2.19–8.06]	3.59 [1.85–6.98]	3.54 [1.82–6.91]	3.41 [1.74–6.68]
*p*-value	< 0.001	< 0.001	< 0.001	< 0.001

*78 students are excluded from the analysis because they have no school bag. OR: odds ratio; **Model 1**: unadjusted; **Model 2**: adjusted for socio-demographic factors (gender, governorate, education of the father); **Model 3**: adjusted for variables in the Model 2 in addition to the weight of the bag, number of days the students carry their bag to schools and number of bags taken to schools; **Model 4**: adjusted for variables in Model 3 in addition to smoking and hours per day spent on watching TV/using computer

## Discussion

This study aimed to estimate the prevalence of LBP amongst adolescents in public high schools in Kuwait, and to examine the association between LBP and the weight of school bag. The data on LBP and its associated factors are scarce in Kuwait, Arab states in the Gulf region and the broader Middle East. We have demonstrated that LBP is common amongst adolescents in public high schools in Kuwait and that the absolute bag weight is not related to LBP.

Approximately, 70% of the adolescents in this study reported having LBP at some point in their life, while 49 and 31% reported having LBP within the last 6 months and within the last month respectively. More than a decade ago, a study in Kuwait showed the lifetime prevalence of LBP amongst junior and high school students to be 57.8% [8]. This estimate seems to be lower than what we report in this study. The difference in the lifetime prevalence between our study and the previous study may reflect a genuine increase in the prevalence of LBP; as the recent literature suggests a trend of increasing LBP prevalence in adolescents, which is now approaching that of adults [16]. However, the difference could also be attributed to the fact that the earlier study included younger adolescents (10 to 18 year) compared to the current study (14 to 19 years) or different methodological approaches. There is also the possibility that the difference could be due to cultural changes that led to an increase in the willingness to report minor LBP, leading to higher acceptance of LBP as an excuse for absenteeism from schools or work [17].

Our study showed that the prevalence of LBP is higher amongst females compared to males. This is consistent with several studies, which showed that young females experience more LBP compared to young males [8–10]. A higher body awareness and perception to pain have been suggested as an explanation for this difference [18]. Furthermore, female menstrual cycle can associate with pain, and may also contribute to the higher prevalence of LBP in female adolescents [18, 19]. In our study, of the 155 females who reported LBP during the last month, 70 thought it could be related to their menstrual cycle. On the contrary, some studies have reported a higher prevalence of LBP in males compared to females, which has been attributed to greater exposure to intense sports and physical activities amongst males [13, 14]. Finally, very few studies have reported similar prevalence of LBP in males and females [20–22].

Several professional associations including the Ontario Chiropractic Association, American Occupational Therapy Association, and American Academy of Pediatrics, have stated that school bag weight should not exceed 10 to 15% of a student’s body weight [12]. Our data does not support such recommendations since bag weight that exceeds 11% or 15% of body weight showed no association with LBP. Other studies also found no association between the relative bag weight and LBP [23, 24], hence do not support these recommendations. In a single study, it was reported that a relative school bag weight > 20% of body weight can double the odds of LBP [25]. Of our study group (950 students), only 19 students had a school bag weight that exceeded 20% of their body weight, which could explain our findings (i.e. relative weight of school bag is not related to LBP). In multivariable analysis, there was no association between LBP and the absolute weight of school bag or the relative weight of the bag (as a percentage of the body weight). This is consistent with other studies that reported no significant association between LBP and the accurately measured weight of school bags in Brazil and in the UK [26, 27]. Also, Jones et al. [24] conducted a cohort study in the UK and showed no association between the weight of school bag and the risk of LBP amongst 11 to 14 year old students.

In our study, the perceived heaviness of school bag (not the accurately measured weight) was significantly associated with LBP throughout the analysis (Table 5). Szpalski et al. [28] and Gunzburg et al. [29] also showed that students who consider their bags to be heavy are more likely to have LBP than other students. In a similar study that measured both the actual bag weight and the perceived heaviness of school bag, the perceived heaviness of school bag (but not the actual bag weight) was significantly associated with LBP [30]. In our study, the association between the perceived heaviness of school bag and LBP can have several explanations. First, since this is a cross-sectional study, it is possible that the students with LBP tend to feel and describe their bag as heavy because of the preexisting pain, regardless of the bag weight (reverse causality). In other words, school bags which are relatively normal in weight are felt heavy and burdensome by students if they have LBP [31]. Second, the higher reporting of bag heaviness amongst students with LBP may be due to their tendency to attribute such pain to a heavy bag [28, 29]. Finally, it is possible that the association between LBP and how the students perceive heaviness of their bag is genuine and that LBP may be the result of what a student considers to be a heavy bag. As such, the perceived heaviness of school bag by students may be a better measure of the impact of the exposure than the actual bag weight, which overlooks many individual factors, such as strength of the muscles, mental preparedness to carry the bag, and many others. In other words, the student is the best judge to define what a heavy bag is for him/her.

In recent years, the weight of school bags has been under intense debate in Kuwait and many other countries. Our data suggests that the perceived heaviness of school bag is far more important than the actual bag weight. The individual student may ultimately be the best judge of how heavy his/her school bag is. Therefore, recommendations on the weight school bag should be based on each student’s judgment on the heaviness of their bag. Parents should be encouraged to ask their children about the heaviness of their school bag and do not allow their children to carry school bags their children describe as heavy. This recommendation would be much easier to implement, as it requires weighing neither the school bag nor the body of the student. Recommendations by professional bodies that are based on relative weight of school bags (as a percentage of body weight) should be revised to take into account the perceived heaviness of the school bag as judged by the student. The current recommendations are not supported by our data or any other data and thus are not evidence-based. One may argue that the association between the perceived heaviness of school bag and LBP (in our studies and other studies) could be explained by reverse causality; and thus does not warrant this discussion. However, even if this is the case, perceived heaviness of the school bag can at least aggravate and prolong LBP and thus should be considered in further recommendations on the weight of school bags. It is well known that the primary and secondary prevention for LBP cannot be separated. Cohort studies are better to address this issue and to investigate if the students who are exposed to heavy bags at an early age are more likely to develop LBP, after taking into account the perceived heaviness of school bag.

This study has several strengths including measuring the weight of school bags, the weight and height of students and the perceived heaviness of school bags. The response rate was high and the data were collected from a nationally representative sample from students in public schools. However, the study has several limitations, one of which is related to the cross-sectional design, which does not allow for causal inference. As mentioned above, the association between the perceived heaviness of school bag and LBP may have risen by reverse causality. It is also possible that students with LBP reduced the weight of their school bag as a result of their LBP. Furthermore, students with severe LBP may have been absent due to their pain, which would underestimate the prevalence of LBP, and also attenuate the association between the weight of school bag and LBP (if it exists). Although we measured the weight of school bags, it is possible that the weight of school bags on the day of data collection does not represent the weight of school bags on other days. This is likely to underestimate the association between the measured weight of school bags and LBP (non-differential misclassification). Finally, we did not gather data on several potential confounders, such as stress [32], and our data cannot be extrapolated to students in private high schools in Kuwait.

## Conclusions

LBP amongst high school students in Kuwait seems to be common with a prevalence resembling that of high-income countries. There was no association between LBP and the absolute weight of school bags neither in univariable nor in multivariable analysis. The relative weight of school bag (as a percentage of body weight) was found to be associated with LBP in univariable analysis but not in multivariable analysis. More importantly, we have demonstrated that the perceived heaviness of school bag is an independent risk factor for LBP. Our findings demonstrate that the perceived heaviness of school bag is far more important than the actual bag weight. Therefore, current recommendations, which are not supported by evidence, should be revised to take into account how students perceive heaviness of their school bags.

## Abbreviations

CI: Confidence Interval
Kg: Kilogram
LBP: Low Back Pain
SD: Standard Deviation
SPSS: Statistical Package for Social Sciences
